# Staging of Alzheimer's disease progression in Down syndrome using mixed clinical and plasma biomarker measures with machine learning

**DOI:** 10.1002/alz.70446

**Published:** 2025-07-19

**Authors:** Mina Idris, Fedal Saini, Phoebe Ivain, R. Asaad Baksh, Leda A. Bianchi, Sarah E. Pape, Henrik Zetterberg, Peter A. Wijeratne, André Strydom

**Affiliations:** ^1^ Department of Forensic and Neurodevelopmental Sciences, Institute of Psychiatry, Psychology & Neuroscience King's College London, London Greater London UK; ^2^ Natbrainlab, Institute of Psychiatry, Psychology & Neuroscience King's College London, London Greater London UK; ^3^ Department of Psychiatry and Neurochemistry, Institute of Neuroscience and Physiology the Sahlgrenska Academy at the University of Gothenburg Mölndal Sweden; ^4^ Clinical Neurochemistry Laboratory Sahlgrenska University Hospital Mölndal Sweden; ^5^ Department of Neurodegenerative Disease UCL Institute of Neurology London UK; ^6^ UK Dementia Research Institute at UCL London UK; ^7^ Hong Kong Center for Neurodegenerative Diseases InnoHK Hong Kong China; ^8^ Wisconsin Alzheimer's Disease Research Center University of Wisconsin School of Medicine and Public Health, University of Wisconsin‐Madison Madison Wisconsin USA; ^9^ Sussex Artificial Intelligence Centre, School of Engineering and Informatics University of Sussex, Falmer Brighton UK

**Keywords:** Alzheimer's disease, blood plasma biomarkers, cognition, cognitive decline, dementia, memory, Down syndrome, event‐based model, machine learning, trisomy 21

## Abstract

**INTRODUCTION:**

Adults with Down syndrome (DS) have a high risk for Alzheimer's disease (AD). Although the sequence of plasma biomarker and cognitive changes in AD in DS is well studied, their related trajectories are not fully characterized. Data‐driven methods can estimate disease progression from cross‐sectional data.

**METHODS:**

In 57 adults with DS and no AD, we used the event‐based model to sequence plasma biomarker and cognitive changes in preclinical AD. Generalized additive models assessed the relationship between age and plasma biomarkers.

**RESULTS:**

The earliest changes occurred in the amyloid beta 42/40 ratio, followed by memory changes. Later alterations in neurofilament light and tau concentrations preceded executive and visuomotor function changes, with glial fibrillary acidic protein levels changing last. The highest rate of plasma biomarker changes occurred between ages 39 and 52.

**CONCLUSION:**

Changes in DS follow a pattern similar to that of sporadic and familial AD. Event‐based modeling offers individual‐level staging, potentially optimizing diagnosis and clinical trial patient selection.

**Highlights:**

- The pre‐clinical stages of Alzheimer's disease (AD) development in Down syndrome (DS) are not well defined, despite the extremely high prevalence of AD.
- Better understanding of early AD progression would aid in diagnostics and treatment.
- Data‐driven methods, such as the event‐based model, can aid in clarifying the sequence of cognitive and plasma biomarker changes in the early stages of AD while accounting for baseline variability.
- We find that plasma amyloid beta 42/40 ratio and memory changes precede changes in plasma biomarker levels of neurodegeneration, with changes in executive and visuomotor functions occurring later, before neuroinflammatory marker changes.
- Combining plasma biomarkers could be a useful measure of preclinical AD for trials, particularly in individuals between 39 and52 years of age.

## 1 BACKGROUND

Down syndrome (DS), caused by a trisomy of chromosome 21, is a genetic disorder characterized by intellectual and physical disabilities and an increased risk of Alzheimer's disease (AD).^1^ This heightened risk is related to the overexpression of the amyloid precursor protein (*APP*) gene found on chromosome 21 and, consequently, an increase in deposition of amyloid beta (Aβ) in the brain. AD pathology often appears by age 40 in DS,^2^, ^3^ decades earlier than the average age of 65 in the general population.^4^, ^5^ In addition, previous research has underscored a compressed timeframe of AD progression in DS, with biomarker and cognitive changes occurring in an accelerated sequence.^6^, ^7^, ^8^

Understanding AD progression in DS requires examining of baseline cognitive abilities and their developmental trajectories. Individuals with DS exhibit a wide range of intellectual disabilities (IDs), and cognitive profiles often include relative strengths in visual processing and social functioning, alongside weaknesses in verbal memory and executive function.^9^, ^10^, ^11^ Researchers have extensively characterized cognitive aging in DS, identifying early signs of cognitive decline, including deficits in memory, attention, and executive function, which may signal AD development.^12^, ^13^, ^14^, ^15^ Cognitive assessment tools tailored to DS, such as the Arizona Cognitive Test Battery and the Cambridge Cognition Examination, have been instrumental in tracking these changes.^16^, ^17^, ^18^ However, despite the known cognitive vulnerabilities in DS, the precise sequence of cognitive decline in relation to biomarker changes remains under study.

Previous literature has shown that cognitive changes in the early stages of AD in DS are linked to abnormal protein deposits in the brain.^19^, ^20^ Proteins related to AD progression can now be measured in plasma, serving as potential biomarkers of disease progression that can be used to track AD‐related degeneration.^21^, ^22^, ^23^ Plasma Aβ42/40 ratio levels decrease in the blood plasma as deposition increases in the brain, whereas increasing plasma neurofilament light (NfL), phosphorylated tau (p‐tau) 181 and 231, and glial fibrillary acidic protein (GFAP) concentrations occurring later provide useful measures of neurodegeneration, tauopathy, and neuroinflammation.^3^, ^24^, ^25^, ^26^ Although there is a strong correlation between plasma biomarkers and cognitive decline in familial and sporadic AD,^27^, ^28^, ^29^ their relationship and the temporal sequence of changes in the early stages of AD in DS remain unclear. Investigating plasma biomarkers of AD in DS and the order in which changes occur can offer useful in vivo insights into disease progression.

Measurements of AD in DS, such as cognitive test scores and plasma biomarkers, are powerful indicators of disease progression and clinical outcomes.^8^, ^21^ However, analysis of these measures is typically limited to one modality, such as different aspects of cognition, or plasma biomarkers, or neuroimaging. Although this approach is useful, it does not adequately reflect the complexities in the progression of AD, which typically affect many modalities and exhibit varying changes in markers years before clinical onset. Incorporating different modalities may provide a more well‐rounded picture of disease progression.

The event‐based model (EBM) is a data‐driven approach to staging disease progression that uses probabilistic methods to disentangle and recognize patterns of changes directly from data, and it can be used to combine results from different measurements to improve accuracy.^20^ These disease progression models can be applied to multimodal cross‐sectional data to estimate disease progression while accounting for baseline variability, and extract information that could aid clinical staging and prognosis. The theory behind this model has been described previously and employed in the study of AD in DS and in other neurodegenerative diseases in the general population.^19^, ^20^, ^30^

Here we aimed to establish the sequence of changes across cognitive performance and blood plasma markers in individuals with DS before the onset of clinical dementia due to AD. We used EBM on cross‐sectional cognitive and AD plasma marker data along the at‐risk age range and aimed to model changes in plasma biomarkers with age, both individually and combined, in order to identify the age range of maximal change.

## 2 METHODS

### 2.1 Study design and exclusion criteria

The London Down Syndrome (LonDownS) study is a longitudinal study of people with DS. All participants undergo extensive neuropsychological examination using the LonDownS Consortium battery, which has been described in previous studies.^8^ The battery involves a series of neuropsychological tests measuring memory, language, executive functions, and motor skills. In addition, a blood sample is collected at the same timepoint as the cognitive assessments.

Inclusion criteria for the wider LonDownS study are individuals aged 16 and older with a confirmed DS diagnosis; the youngest participant included in this study is 20 years of age. For inclusion in this analysis, participants were required to have no diagnosis of dementia at baseline.^31^ Dementia status was established with blind clinical rating by a clinician based on the participant's development of dementia symptoms reported by caregivers using version II of the Cambridge Examination for Mental Disorders of Older People with Down Syndrome and Others with Intellectual Disabilities (CAMDEX‐DS‐II). Individuals with dementia were excluded to focus specifically on pre‐dementia changes.

ID level was defined according to the International Classification of Diseases, Tenth Revision (ICD‐10) diagnostic system and based on parental/caregiver report of the individual's best level of functioning according to the general functional abilities associated with IQ levels: mild (50 to 69), moderate (35 to 49), and severe/profound (<35) ID.^32^

Participants in this analysis were categorized into two age groups: younger adults (YA) ages 20–35 years and older adults (OA) ages 36–59. YA were classified as the pre‐decline “control” group and considered to be cognitively stable and at their cognitive peak, with minimal AD‐related biomarker and cognitive changes. In contrast, OA were classified as the preclinical “neuropathological” group, as research suggests that AD‐related neuropathology, such as amyloid deposition and other biomarker changes, begins to emerge in individuals with DS around their mid‐30s.^1^, ^20^, ^33^ This threshold was selected to better capture the early stages of AD‐related biomarker and cognitive changes to compare between a cognitively stable group and an at‐risk group within the preclinical window of AD progression.

**RESEARCH IN CONTEXT**

1. **Systematic review**: A literature review was conducted using the PubMed database. People with Down syndrome (DS) have an extremely high risk of developing Alzheimer's disease (AD) as they age. Despite this, little is known about the markers of the earliest stages of AD and progression in DS. Moreover, there is limited research on measures that are sensitive to preclinical changes.
2. **Interpretation**: We showed that a multimodal data‐driven method can provide insights into early AD‐related changes using common tests of cognition and plasma biomarkers of AD. More knowledge on the order of changes in preclinical stages of AD can allow for diagnoses and potential disease‐modifying therapies to be trialed at an earlier, more targeted stage.
3. **Future directions**: Future work should incorporate imaging data, informant ratings of symptom development, and longitudinal data to develop the model and add a temporal aspect whereby time between disease‐related changes can be estimated.

### 2.2 Cognitive outcome measures

We selected measures from the LonDownS cognitive battery that have been proven previously to be feasible and sensitive to AD progression in DS.^14^, ^34^, ^35^ These cognitive measures showed good psychometric properties, are suited to a range of ages and abilities, including non‐verbal individuals, and have good test–retest reliability across the age groups.^16^, ^19^ Cognitive tasks included for this analysis were:

1. Memory: Cambridge Neuropsychological Test Automated Battery Paired Associates Learning (CANTAB PAL) first trial memory score.^36^
2. Executive functioning, working memory, and planning: Tower of London total score.^37^, ^38^
3. Rule learning and set shifting: CANTAB Intra/Extra Dimensional Set Shift (CANTAB IED) number of stages completed.^36^
4. Visuomotor skills, hand‐eye coordination: Developmental NEuroPSYchological Assessment, Second Edition—visuomotor precision (NEPSY‐II) car and motorcycle score.^39^

Please see Table S1 for detailed descriptions of these tasks and outcome measures.

### 2.3 Plasma biomarker measures

Venipuncture of the antecubital fossa was performed on the same day as cognitive testing. Blood plasma samples were collected in ethylenediaminetetraacetic acid (EDTA) tubes (Fisher Scientific UK, Loughborough, UK) for biomarker analysis. Plasma Aβ40, Aβ42, p‐tau181, NfL, and GFAP concentrations (pg/mL) were measured using commercially available assays on a single molecule array (Simoa) HD‐X instrument according to protocols issued by the manufacturer (Quanterix). Plasma p‐tau231 concentration was measured using an in‐house Simoa assay, as described previously.^40^

All measurements were performed in one round of experiments with one batch of reagents. Intra‐assay coefficients of variation were below 10%. Outliers in plasma biomarker measurements, defined as values exceeding 1.5 times the interquartile range, were excluded from this analysis to minimize the influence of anomalous data and to ensure that the EBM was valid. Specifically, there were four outliers for Aβ42/40 ratio, three for p‐tau181, and two each for p‐tau231 and GFAP. Retaining these outliers affected the ordering of Aβ42/40 ratio by the EBM.

### 2.4 Descriptive statistics

Descriptive statistics were used to summarize the sample and its characteristics. Kruskal–Wallis tests, controlling for ID level, were performed to determine which measures were significantly different between the younger and older groups. Statistical significance was set at *p *< 0.05. All statistical analyses and EBM were performed using Python 3.11.7^41^ in JupyterLab 4.0.11.^42^

### 2.5 Event‐based model (EBM)

The EBM is an unsupervised mixture modeling technique that can learn the order in which biomarkers transition from a normal to abnormal state (an event). The EBM has previously demonstrated its ability to differentiate between heterogeneous inputs, such as the YA and OA groups used here.^4^, ^13^ The YA and OA groups were used as the control and disease groups for the model, respectively. Measures from the cognitive tests and plasma biomarker analysis (adjusted for ID level) were used as inputs for the EBM. To distinguish between baseline cognitive deficits related to ID and cognitive decline related to or preceding dementia development, ID level (mild and moderate) was included as a covariate by estimating residuals in the YA group for each marker based on linear regression coefficients, after which these coefficients were used to calculate residuals for all individuals’ measurements.

The EBM analysis uses Markov Chain Monte Carlo (MCMC) sampling to estimate the sequence of events by sampling data and simulating many possible event sequences. This generates a posterior distribution of likely event orders, providing probabilities for different event sequences. The model sequence is visualized as a positional variance diagram, which shows the most likely estimated sequence of marker events, with sampling uncertainty. This estimated event order reflects the order of changes in cognitive measures and plasma biomarkers transitioning from a pre‐decline range to decline associated with AD.^30^, ^43^

A secondary output of the EBM analysis is a disease stage for each individual, which corresponds to their most likely point along the positional variance event sequence. Each participant is assigned an estimated EBM disease stage based on the cognitive and plasma biomarker data and the predicted event sequence. The histogram shows the distribution of events across the two age groups, illustrating at which EBM stages these events are most likely to occur for each group. Increased EBM stage is a strong predictor of conversion to AD.^44^

### 2.6 Modeling biomarker trajectories with age

Plasma concentrations of the Aβ42/40 ratio, p‐tau218 and 231, GFAP, and NfL were standardized to zero mean and unit variance to remove scale differences prior to principal component analysis (PCA). Missing biomarker data were handled using multiple imputation by the mice package in R for PCA. Ten imputed datasets were generated using predictive mean matching with 10 iterations, incorporating all relevant biomarkers and predictors to inform the imputation. After imputation, biomarker values were log‐transformed, and the Aβ42/40 ratio was computed within each imputed dataset. Subsequent analyses were conducted separately on each imputed dataset, and results were pooled across imputations following Rubin's rules to appropriately account for uncertainty due to missing data.

PCA was then applied to the standardized biomarker data to identify the primary patterns of variation. Two principal components (PCs) were retained, collectively explaining a substantial proportion of the total variance. Component loadings were examined to assess the relative contribution of each plasma biomarker to the identified components.

To model the association between age and the derived PCs, generalized additive models (GAMs) were fitted. GAMs were chosen to flexibly capture potential non‐linear relationships between age and the multivariate biomarker profiles represented by the PCs. GAMs were also fitted on the raw plasma biomarker data.

As a supplementary sensitivity analysis, a locally estimated scatterplot smoothing (LOESS) analysis was conducted. Biomarker concentrations for plasma Aβ42/40 ratio, p‐tau, GFAP, and NfL were standardized into *z*‐scores to normalize the data and facilitate comparisons and combinations across biomarkers with differing scales. LOESS regression was implemented using Python 3.11.7^41^ in JupyterLab 4.0.11^42^ to model the smoothed relationships between age (independent variable) and *z*‐scored biomarker levels (dependent variable). Separate LOESS curves were generated for each plasma biomarker.

Finally, an additive model was used to evaluate the impact of overall biomarker burden, whereby individual biomarker *z*‐scores for plasma Aβ42/40 ratio, p‐tau, GFAP, and NfL were summed to create a composite *z*‐score for each participant to identify the age range within which most change occurs before dementia diagnosis. The *z*‐score for the Aβ42/40 ratio was inverted in this additive model to align in the direction of pathology across all plasma biomarkers. The precision of the LOESS estimates was assessed by calculating 95% confidence intervals (CIs) around the smoothed regression line. CIs were derived from the residuals of the LOESS fit, accounting for variability within the data and uncertainty in the model predictions.

## 3 RESULTS

### 3.1 Participants

**FIGURE 1:**
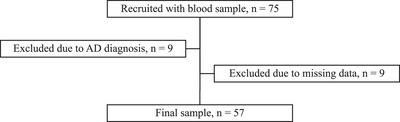
Flowchart illustrating the participant exclusion process and the final sample size.

From the total number of individuals with blood samples recruited for LonDownS at the time of completing the assays (*n* = 75), 18 were excluded from the analyses because they presented with an AD diagnosis at assessment or 75% of their cognitive data was missing (Figure 1). The final sample consisted of 57 adults with DS, evenly split into YA (*n* = 29, mean age = 26.70 years) and OA (*n* = 28, mean age = 48.39 years) groups. The total sample had a mean age of 37.35 years (range: 20–59). Male participants comprised 59.65% of the sample. Most participants were White (89.47%), with smaller representations of Black (5.26%), Asian (3.51%), and Other (1.75%) ethnicities. In terms of ID, 47.37% had mild ID, whereas 52.63% had moderate ID. The OA group had a higher proportion of individuals with mild ID compared to the YA group (see Table 1 for the demographic characteristics of the final sample).

**TABLE 1:** Demographic data of younger and older adults by age group.

Demographics	Younger adults	Older adults	Total sample
**Total sample, *n* (%)**	29 (50.88)	28 (49.12)	57 (100.00)
**Age**			
Mean	26.70	48.39	37.35
Standard deviation	4.11	6.65	12.23
Range	20–35	36–59	20–59
**Sex, *n* (%)**			
Female	12 (41.38)	11 (39.29)	23 (40.35)
Male	17 (58.62)	17 (60.71)	34 (59.65)
F:M ratio	12:17	11:17	23:34
**Ethnicity, *n* (%)**			
White	25 (86.21)	26 (92.86)	51 (89.47)
Black	1 (3.45)	2 (7.14)	3 (5.26)
Asian	2 (6.90)	0 (0)	2 (3.51)
Other	1 (3.45)	0 (0)	1 (1.75)
**Level of ID, *n* (%)**			
Mild	11 (37.93)	16 (57.14)	27 (47.37)
Moderate	18 (62.07)	12 (42.86)	30 (52.63)

Abbreviation: F, female; ID, intellectual disability; M, male.

### 3.2 EBM inputs

Kruskal–Wallis tests were performed controlling for ID level, and raw marker measures with a statistically significant difference between the YA and OA groups were used as input for the EBM. See Table 2 for descriptive statistics and Kruskal–Wallis test results of plasma biomarkers and cognitive markers.

**TABLE 2:** Comparing plasma biomarker concentrations (pg/mL) and cognitive task performance: Mean and (standard deviation) between younger and older adults.

Marker	Younger adults	Older adults	Total *n*	*p*‐value
Aβ42/40	0.06 (0.01)	0.06 (0.01)	53	0.058
p‐Tau181	4.01 (1.94)	7.02 (3.31)	54	0.000
p‐Tau231	5.41 (1.38)	9.38 (3.67)	55	0.000
NfL	8.79 (4.79)	17.06 (5.93)	57	0.002
GFAP	66.74 (26.09)	100.65 (43.39)	55	0.000
PAL first trial memory score	11.17 (4.67)	6 (5.06)	48	0.000
Tower of London	7.44 (2.82)	5.42 (3.59)	53	0.024
NEPSY‐II car and motorbike	18.86 (8.31)	11.07 (8.32)	55	0.000
IED stages completed	7.15 (1.83)	5.22 (3.00)	50	0.003

Abbreviations: Aβ, amyloid beta; CANTAB IED, Cambridge Neuropsychological Test Automated Battery Intra/Extra Dimensional Set Shift; CANTAB PAL, Cambridge Neuropsychological Test Automated Battery Paired Associates Learning; GFAP, glial fibrillary acidic protein; NEPSY‐II, Developmental NEuroPSYchological Assessment, Second Edition; NfL, neurofilament light; p‐tau, phosphorylated tau.

### 3.3 Event sequencing

**FIGURE 2:**
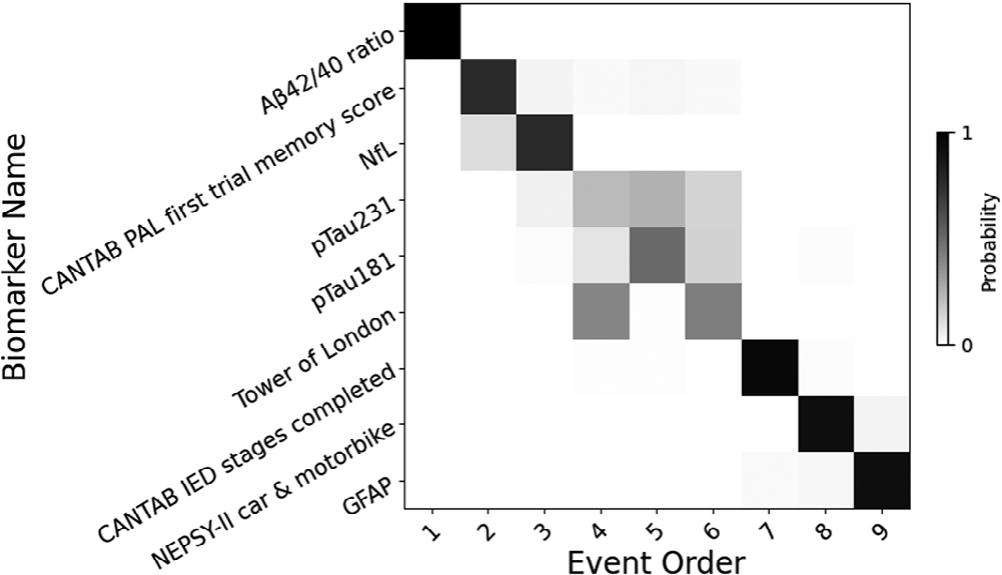
Event‐based model positional variance diagram. This shows the estimated order of cognitive and blood plasma marker abnormality events in this DS cohort. The heatmaps indicate the magnitude of the uncertainty of the ordering; dark diagonal boxes indicate strong event ordering with less uncertainty, and lighter indicate possible event permutations with strength proportional to the off‐diagonal boxes. Aβ, amyloid beta; DS, Down syndrome; GFAP, glial fibrillary acidic protein; CANTAB IED, Cambridge Neuropsychological Test Automated Battery Intra/Extra Dimensional Set Shift; NEPSY‐II, Developmental NEuroPSYchological Assessment; NfL, neurofilament light; CANTAB PAL, Cambridge Neuropsychological Test Automated Battery Paired Associates Learning; p‐tau, phosphorylated tau.

An EBM was fit using the YA as controls and the OA group as the disease population, and a maximum‐likelihood event sequence was obtained together with sampling uncertainty (Figure 2). The EBM found that the earliest changes are in blood plasma markers Aβ42/40 ratio and NfL levels, coinciding with cognitive declines in memory (CANTAB PAL). This was followed by changes in p‐tau biomarkers and executive function (Tower of London and CANTAB IED) in people with DS. Finally, visuomotor decline (NEPSY‐II) presented as the latest cognitive event along with changes in plasma GFAP concentrations.

### 3.4 Disease staging

**FIGURE 3:**
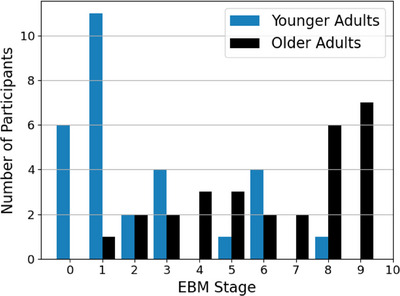
Disease staging predicted by the event‐based model sequence, colored by age group.

Using the maximum‐likelihood event sequence in the positional variance diagram, each participant was assigned an EBM disease stage. The distribution of stages in the YA and OA groups (Figure 3) showed that the YA group was substantially more likely to be at an earlier stage of disease (including stage 0), experiencing earlier events as set out by the EBM positional variance diagram. The OA group, made up of individuals who are all assumed to have some degree of AD neuropathology, showed a spread along the event stages, with many at later disease stages.

### 3.5 Principal component analysis

Following multiple imputation, PCA was conducted on the five standardized plasma biomarkers: Aβ42/40 ratio, p‐tau181, p‐tau231, GFAP, and NfL. Two PCs were extracted from the pooled average loadings, cumulatively accounting for 77.4% of the total variance (PC1: 56.5%; PC2: 20.9%).

The first principal component (or PC1) loaded positively on p‐tau181 (0.528), p‐tau231 (0.499), GFAP (0.484), and NfL (0.481), suggesting that i reflects a general neurodegeneration and tauopathy‐related biomarker pattern. The second component (or PC2) was strongly and uniquely associated with the Aβ42/40 ratio (0.941), indicating that PC2 primarily captures amyloid‐related variance (Table 3).

**TABLE 3:** Pooled average loadings of plasma biomarkers on the first two principal components (PC1 and PC2) derived from principal component analysis conducted across multiple imputed datasets.

Plasma biomarker	PC1	PC2
Aβ42/40 ratio	0.077146	0.940641
p‐Tau181	0.527525	−0.264592
p‐Tau231	0.499133	−0.106737
GFAP	0.483528	0.122381
NfL	0.481131	0.126699

*Note*: These loadings highlight the relative contributions of Aβ42/40 ratio, p‐tau181, p‐tau231, GFAP, and NfL to the variance in AD‐related biomarker concentrations.

Abbreviations: Aβ, amyloid beta; AD, Alzheimer's disease; GFAP, glial fibrillary acidic protein; NfL, neurofilament light; p‐tau, phosphorylated tau.

### 3.6 Generalized additive models

**FIGURE 4:**
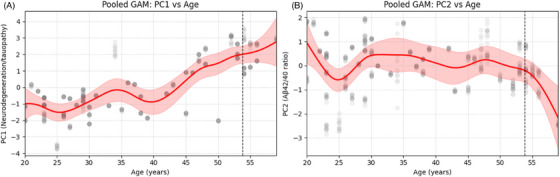
GAM fits showing the association between age and the first two PCs derived from plasma biomarkers pooled across 10 imputed datasets. (A) PC1 represents a composite of neurodegeneration‐ and tauopathy‐related markers. (B) PC2 primarily captures variance related to Aβ42/40 ratio, with minimal contributions from other biomarkers. Red lines indicate the pooled fitted GAM smooths, representing the estimated mean trajectory of each component with age. The shaded red areas denote the 90% CIs around these smooths, which were calculated using Rubin's rules to account for uncertainty across the multiple imputations. Gray dots represent individual participant scores for PC1 and PC2 across all imputed datasets. The vertical dashed black line at age 53.8 years denotes the average age at AD onset in DS, based on prior literature, and serves as a reference point. AD, Alzheimer's disease; Aβ, amyloid beta; CI, confidence interval; DS, Down syndrome; GAM, generalized additive model; PC, principal component.

To examine age‐related trends in plasma biomarker profiles, GAMs were fitted to the first two PCs derived from the standardized biomarker data (Figure 4). PC1, which primarily reflects neurodegeneration‐ and tauopathy‐related markers, showed a non‐linear increase with age (Figure 4A). Although PC1 values remained relatively stable from age 20 to ≈39, a noticeable upward trend emerged after age 39, with an accelerated increase approaching the mean age at AD onset. PC2, which was driven largely by variation in the Aβ42/40 ratio, demonstrated a decline across age (Figure 4B).

Figure S1 shows GAM fits for the raw plasma biomarker concentrations and age. Aβ42/40 ratio exhibited a decline across the age range, particularly after age 50. In contrast, p‐tau181 and p‐tau231 demonstrated marked nonlinear increases beginning around age 45–50, suggestive of age‐associated tauopathy. GFAP followed a similarly increasing trend, with a peak at around age 50, whereas NfL showed a more gradual increase with age from age 30.

### 3.7 LOESS analysis

Figure S2A shows that the Aβ42/40 ratio changed at around age 25, followed by a steady decrease thereafter. In contrast, in Figure 2C,F, NfL and GFAP increased steadily with age from age 30, ≈20 years before the mean age at AD diagnosis (53.8 years) in DS.^2^ Figure 2D and shows that p‐tau231 and p‐tau181 levels are relatively stable and begin to rise from age 39, showing the highest increase in biomarker levels. In the composite models (Figure 2G,H), combined biomarkers show a consistent increase in summed plasma biomarker *z*‐scores from age 30 until the average age at diagnosis, with the highest rate of change between ages 39 and 52.

## 4 DISCUSSION

### 4.1 Main findings

This study applied a disease progression model, the EBM, to characterize the sequence of cognitive and plasma biomarker changes in adults with DS without clinical AD. This represents the first time the EBM has been used to analyze this combination of markers in DS, offering insights into preclinical AD progression in this population.

Our findings suggest that, in DS, early AD‐related cognitive and blood marker changes, despite occurring at a younger age, follow patterns similar to those observed in the general population.^6^, ^20^ The EBM confirmed that plasma amyloid changes occur early, followed by changes in memory and biomarkers of tauopathy and neurodegeneration. Later changes were observed in executive functioning and visuomotor ability, and finally, in neuroinflammatory markers.

GAM and LOESS regression identified a critical window between 39 and 52 years when plasma biomarkers showed maximal change. This age range may be particularly important for early detection and intervention. Insights into the sequence of changes and crucial age ranges can guide the selection of participants and biomarkers for clinical trials aimed at early AD stages in DS.

### 4.2 Staging of cognitive and plasma biomarker changes

The EBM found that changes in plasma Aβ42/40 ratio precede tauopathy (p‐tau231, p‐tau181), neurodegeneration (NfL), and neuroinflammation (GFAP).^21^, ^25^, ^45^ This reflects patterns observed in familial and sporadic AD, where Aβ accumulation is an early hallmark.^28^, ^33^ Although plasma Aβ42/40 ratio may not be a strong predictor of cognitive decline due to changes in this marker preceding cognitive decline by several decades, it remains valuable for early detection.^26^, ^33^

Memory decline (assessed by CANTAB PAL) followed changes in plasma Aβ42/40 ratio, likely reflecting increased amyloid deposition to the hippocampus and medial temporal lobe.^46^, ^47^, ^48^ This is consistent with findings in the general population, where Aβ42/40 ratio changes are associated with, and followed by, memory and visuospatial function decline.^28^

Subsequent increases in plasma NfL, p‐tau231, and p‐tau181 indicate ongoing early tauopathy and neurodegeneration, supporting their utility as progression markers.^24^, ^45^, ^49^ In the general population, elevated p‐tau181, p‐tau231, and NfL levels predicted faster cognitive decline, and strong relationships were found between increased p‐tau231 and declines in executive function and attention.^25^ This is consistent with EBM findings in DS, where changes in p‐tau231 and p‐tau181 precede changes in measures of executive functioning, as assessed by the Tower of London and CANTAB IED.^28^

Later impairments in visuomotor precision and attention (measured by NEPSY‐II) emerged before the final changes in plasma GFAP concentrations. Previous studies found GFAP to be a potential prognostic biomarker, correlating with cognitive decline. We found that all cognitive domains had shown changes before GFAP levels entered a measurably abnormal range.^27^ This sequence of cognitive decline and biomarker changes supports the hypothesis that DS‐related AD affects individuals in a predictable order, similar to that seen in sporadic and genetic AD.^6^, ^20^, ^48^, ^50^

### 4.3 Age‐related trajectories of plasma biomarkers

GAMs complement the EBM analysis by modeling the non‐linear trajectories of plasma biomarkers with age, revealing inflection points preceding symptom onset. PC1, driven by NfL, p‐tau181, p‐tau231, and GFAP, suggests age‐related increases in neurodegeneration, tau pathology, and neuroinflammatory markers, respectively.

We demonstrated that p‐tau231 and p‐tau181 levels began increasing at approximately age 39, 15 years before average clinical diagnosis at age 53.5. These trajectories align with those reported in sporadic, genetic, and DS AD, where plasma p‐tau begins increasing 15 years prior to familial AD onset.^6^, ^20^, ^29^, ^48^, ^50^ Our GAM and LOESS findings also indicate a prominent increase in GFAP beginning at age 36, which is somewhat earlier than previous studies but nonetheless in keeping with other reports of GFAP levels.^3^, ^51^

PC2, driven by Aβ42/40 ratio, showed early decline from age 25, followed by relative stability until a pronounced decline from age 50. This pattern, supported by the EBM, LOESS regression, and prior studies,^52^ is consistent with early amyloid deposition. Plasma Aβ42/40 ratio decline with age is a common early hallmark of AD, even before clinical symptoms appear, due to reduced Aβ42 clearance or increased aggregation into neural amyloid plaques.^12^, ^33^

In contrast, NfL, p‐tau, and GFAP rose more markedly in the decade preceding clinical onset, consistent with the amyloid cascade hypothesis. The most pronounced combined biomarker increases occurred between ages 39 and 52 years, highlighting a potential preclinical window of pathological change prior to AD onset in DS.^6^, ^23^, ^53^ These results support multivariate methods like PCA and GAMs for capturing dynamic biomarker changes and improving early disease modeling. Composite biomarker strategies can enhance early detection, improve predictive accuracy, and reduce clinical trial durations.^54^, ^55^, ^56^, ^57^

### 4.4 Integration of biomarker trajectories within the ATN framework

Consistent with the amyloid/tau/neurodegeneration (ATN) framework, we observed early amyloid pathology reflected by reductions in the plasma Aβ42/40 ratio, which aligns with the well‐established amyloid hypothesis.^53^ However, unlike the sequence proposed by Jack et al. for sporadic AD and by Fortea et al. for DS, the findings here suggest that markers of neurodegeneration and glial activation (NfL, GFAP) are in keeping with meta‐analyses showing their association with disease progression, and that they may rise concurrently with, or slightly prior to, detectable increases in tau pathology.^6^, ^53^, ^58^

Notably, although plasma NfL and p‐tau markers show measurable abnormality before GFAP in the EBM, this does not necessarily indicate that GFAP changes occur later. The EBM determines event ordering based on when biomarker levels cross statistical abnormality thresholds relative to the sample distribution, and therefore earlier but subtler changes in GFAP may precede p‐tau changes (as seen in the GAMs) without being classed as an event until later.

### 4.5 Implications for clinical trials

Given the early onset and the high prevalence of AD neuropathology in DS, this population is crucial for trialing interventions aimed at slowing or preventing disease progression.^19^ Despite this, historically, clinical trials in DS have been limited by a lack of reliable outcome measures and longitudinal data.^59^ However, as more people with DS are included in trials, our findings demonstrate that combining plasma biomarkers and cognitive tests can effectively stage AD progression in DS.

Our results suggest that the EBM is effective at staging disease progression by assigning individuals a likely disease stage based on their data, with findings comparable to the previous literature. This further highlights the potential of EBM in identifying early disease changes and supports earlier, targeted interventions to improve outcomes. It is important to note that we have confirmed that the earliest changes occur in the plasma Aβ42/40 ratio, with a decline as early as age 25, ≈30 years earlier than the average AD diagnosis in DS.

Clinical trials aiming to prevent AD progression before clinical changes could consider including a combined plasma biomarker outcome and prioritizing recruitment of individuals within or just before the 39 to 52 age range, where biomarker changes are most pronounced. This would likely capture the highest expected rate of pathological change and could inform sample size considerations and overall trial design.^19^ Moreover, shifting from traditional cerebrospinal fluid biomarkers to plasma biomarkers in the early stages of AD in DS, as suggested by previous research in the general population, would be more cost‐effective and less invasive. This could improve clinical trial feasibility in DS.^15^, ^52^, ^60^, ^61^

### 4.6 Limitations and future directions

The primary strength of this study is the integration of plasma biomarkers with cognitive measures, offering a more comprehensive view of AD progression in DS. However, the cross‐sectional design and a relatively modest sample size limit inferences about timings between disease stages and rates of change.

Future studies with larger sample sizes will explore advanced modeling techniques including temporal EBM and subtyping, such as Subtype and Stage Inference, on cross‐sectional and longitudinal data, to estimate time intervals, calculate rates of change, and reveal distinct AD progression subtypes in DS. This would provide a better understanding of disease progression, thus enabling more personalized treatment and improving clinical trial design by highlighting variability in disease progression.^62^, ^63^

In addition, although we utilized cognitive tests that are sensitive to AD changes in DS, incorporating neuroimaging and informant‐rated behavioral and functional measures would also provide a more comprehensive view of the disease's course.^64^, ^65^ This is key for understanding the early stages of AD, as previous research reports that, unlike in the general population, behavioral and personality changes may be reported at earlier stages of AD in DS.^50^, ^66^

Although diverse in age and gender, this sample lacked representation from Black and Asian groups. In addition, although the broader research study includes individuals with severe ID, this analysis was limited to participants with mild or moderate ID who could complete cognitive testing, potentially affecting the generalizability of the findings. Future studies should include a more diverse and representative sample.

## 5 CONCLUSIONS

This study provides valuable insights into AD progression in DS by combining plasma and cognitive measures. Findings show similarities with sporadic and genetic AD, with early changes in Aβ levels and memory, followed by neurodegenerative plasma biomarkers and further cognitive and visuomotor decline. Mapping changes and age ranges in which individuals are most affected provides valuable groundwork for early diagnosis and intervention.

## AUTHOR CONTRIBUTIONS

The authors confirm their contribution to this work as follows. M.I., P.A.W., and A.S. contributed to the conception and design of the study. F.S. and S.E.P. collected the data. H.Z. conducted the laboratory plasma biomarker analyses. M.I., L.A.B., R.A.B., S.E.P., and P.I. processed the data. M.I. analyzed the data with supervision and support from P.A.W. M.I. wrote the manuscript with input from all authors. All authors contributed to the article, and read and approved the submitted manuscript.

## CONFLICT OF INTEREST STATEMENT

H.Z. has served on scientific advisory boards and/or as a consultant for Abbvie, Acumen, Alector, Alzinova, ALZpath, Amylyx, Annexon, Apellis, Artery Therapeutics, AZTherapies, Cognito Therapeutics, CogRx, Denali, Eisai, Enigma, LabCorp, Merry Life, Nervgen, Novo Nordisk, Optoceutics, Passage Bio, Pinteon Therapeutics, Prothena, Quanterix, Red Abbey Labs, reMYND, Roche, Samumed, Siemens Healthineers, Triplet Therapeutics, and Wave; has given lectures sponsored by Alzecure, BioArctic, Biogen, Cellectricon, Fujirebio, Lilly, Novo Nordisk, Roche, and WebMD; and is a co‐founder of Brain Biomarker Solutions in Gothenburg AB (BBS), which is a part of the GU Ventures Incubator Program (outside submitted work). A.S. received funding from AC Immune and served on advisory boards for ProMIS neurosciences, AC Immune, and Acta Pharmaceuticals. The other authors declare that they have no competing interests. Author disclosures are available in the supporting information.

## ETHICS APPROVAL AND CONSENT TO PARTICIPATE

The authors assert that all procedures contributing to this work comply with the ethical standards of the relevant national and institutional committees on human experimentation and with the Helsinki Declaration of 1975, as revised in 2008. All procedures involving human subjects were approved by the North‐West Wales Research Ethics Committee (13/WA/0194). Capacity was evaluated for participants at each assessment, and written informed consent was obtained from all participants where possible. For those participants who lacked capacity, their carers acted as consultees. Carers were required to indicate their decision about the participants’ inclusion based on their knowledge of the participant and their wishes, in accordance with the UK Mental Capacity Act 2005.

## DIVERSITY, EQUITY, AND INCLUSION STATEMENT

This research addressed diversity, equity, and inclusion through:

- Study design: Recruitment through the NHS to reach a wide range of individuals across the UK and a diverse participant sample. The study was open to all adults with Down syndrome (DS), regardless of levels of intellectual disability (ID), language ability (including non‐verbal), or consent capacity.
- Execution: Flexible data collection accommodated participants’ needs and ensured equitable participation. This included flexible scheduling, covering transportation and food costs, using culturally sensitive protocols, and involving staff trained in ID and culturally competent research practices.
- Interpretation: The data analysis did not explicitly account for the diversity of the sample or examine outcomes across different demographic groups. Potential disparities in the findings based on factors such as sex, race, or ethnicity were not directly assessed. Future studies should aim to explore these factors in more detail to better understand how they might influence the results.

Despite these efforts, the study sample lacked adequate representation from certain demographic groups, including those from non‐White British backgrounds. This is acknowledged in the limitations section of the discussion. By addressing these aspects, we aim to promote inclusivity and equity in research and recognize the importance of diverse representation in advancing scientific knowledge.

## Supporting information



Supporting Information

Supporting Information

Supporting Information

Supporting Information

## Data Availability

The datasets used and analyzed in the current study are available from the corresponding author on reasonable request.
