# Supplementary material for: Staging of Alzheimer's disease progression in Down syndrome using mixed clinical and plasma biomarker measures with machine learning
Source: Alzheimers Dement. 2025 Jul 19;21(7):e70446. doi: 10.1002/alz.70446 (PMC12276070; doi:10.1002/alz.70446)
Supplement: Supplementary file 2 — Supporting Information [file ALZ-21-e70446-s003.pdf]

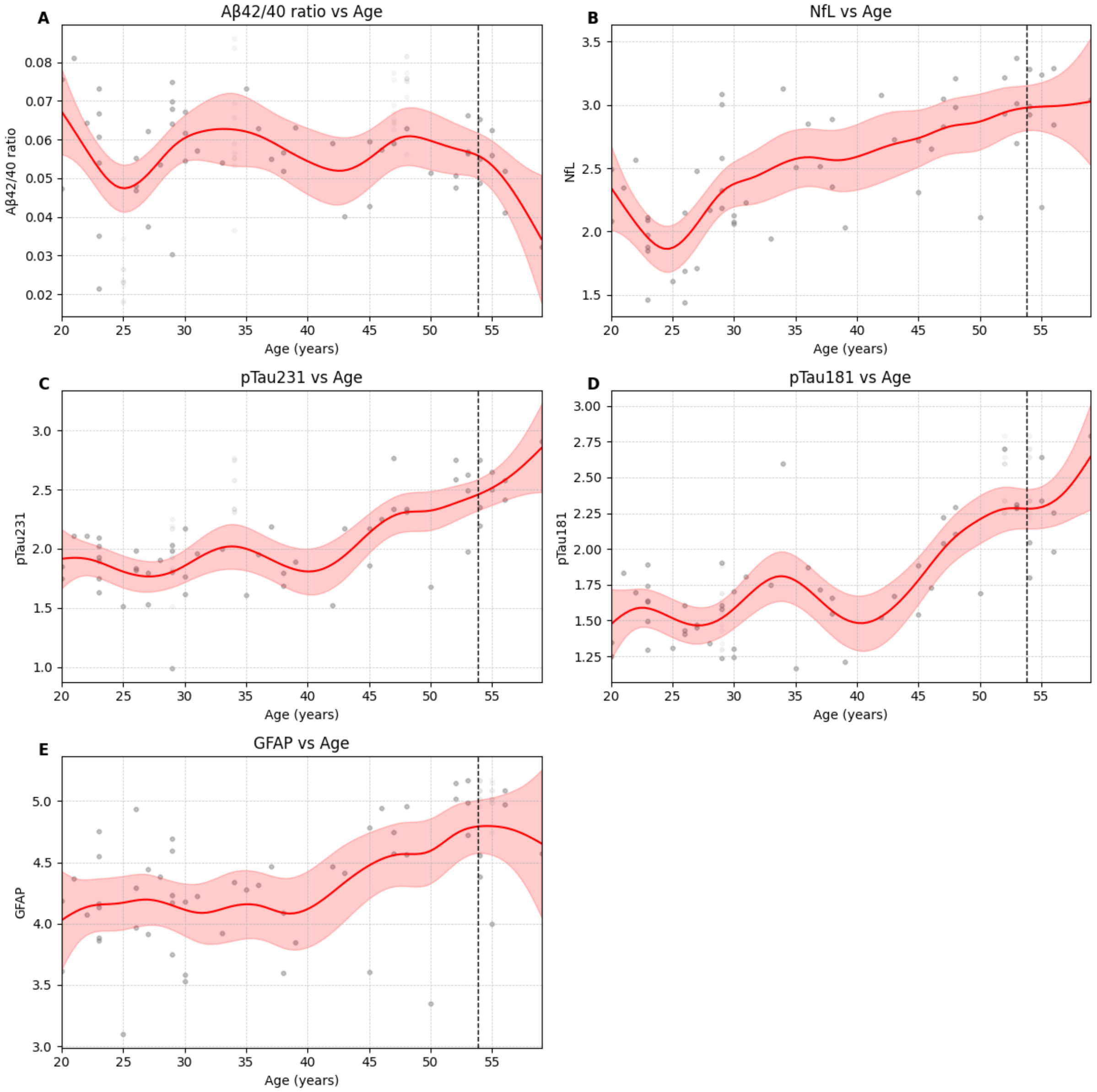

Supplementary Figure 1. Generalised Additive Models (GAMs) of the association between age and standardised AD-related plasma biomarkers. Green lines represent the pooled GAM smooths across 10 imputed datasets, with shaded areas indicating the 95% confidence intervals. Grey dots represent individual participants from all imputations. The vertical dashed black line at age 53.8 years marks the average age of AD onset in individuals with DS.

Note: Aβ = amyloid-beta, NfL = neurofilament light, pTau = phosphorylated tau, GFAP = glial fibrillary acidic protein.
