# Supplementary material for: Staging of Alzheimer's disease progression in Down syndrome using mixed clinical and plasma biomarker measures with machine learning
Source: Alzheimers Dement. 2025 Jul 19;21(7):e70446. doi: 10.1002/alz.70446 (PMC12276070; doi:10.1002/alz.70446)
Supplement: Supplementary file 3 — Supporting Information [file ALZ-21-e70446-s001.pdf]

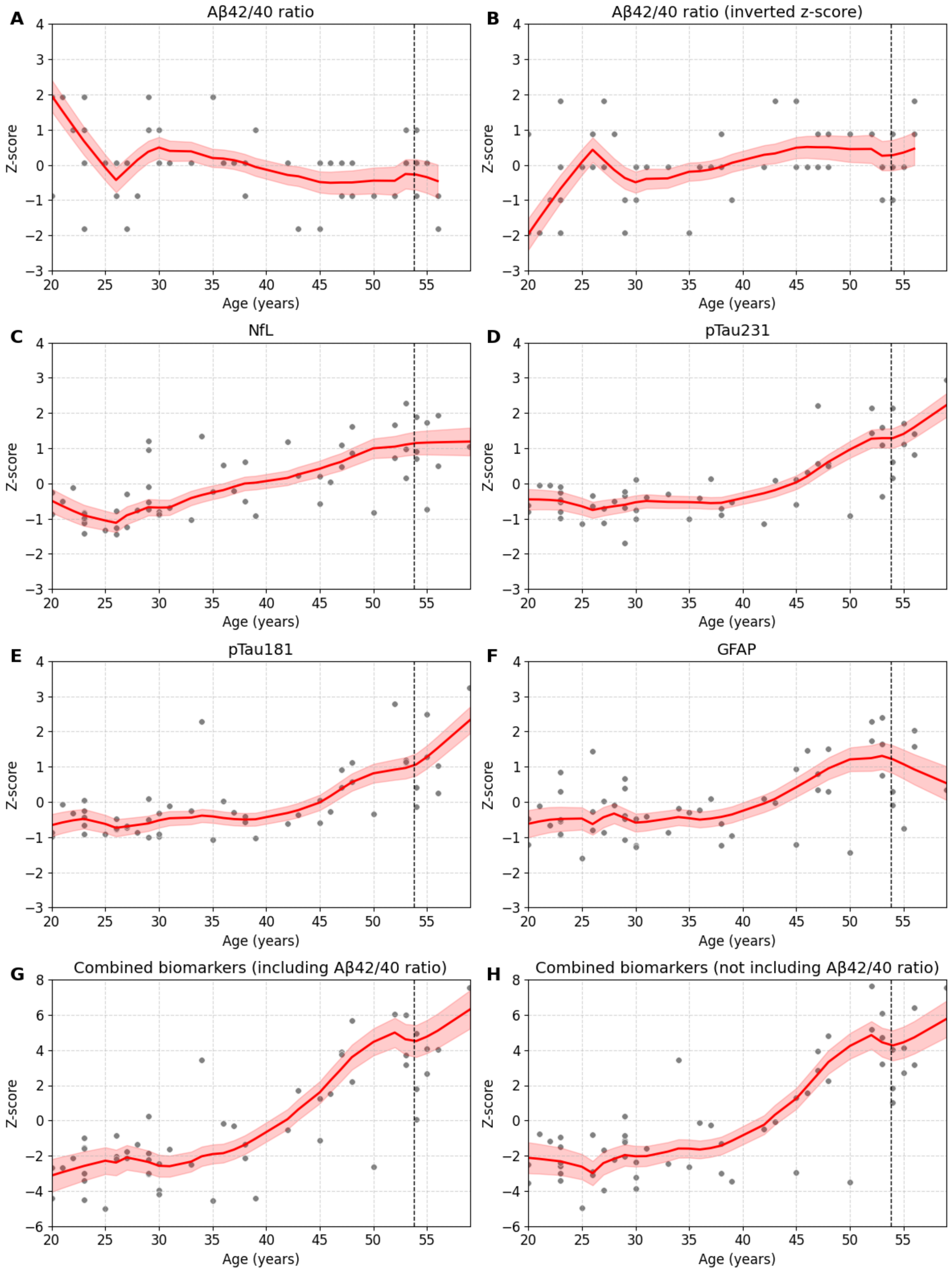

Supplementary Figure 2. Relationship between age and AD-related plasma biomarker z-scores, modelled using LOESS regression. The red curve represents the smoothed LOESS fit, with the shaded area indicating the 95% confidence interval. The black vertical dashed line at

age 53.8 years denotes the average age of AD onset, based on prior literature, and serves as a reference point.

Figure 2B displays the inverted Aβ42/40 ratio z-score included in the additive model in **Figure 2G**. The z-score for Aβ42/40 ratio was inverted in this additive model to align in the direction of pathology across all plasma biomarkers.

Figure 2H shows an additive model of all the biomarkers excluding the Aβ42/40 ratio.

Note: Aβ = amyloid-beta, NfL = neurofilament light, pTau = phosphorylated tau, GFAP = glial fibrillary acidic protein.
