# Supplementary material for: Staging of Alzheimer's disease progression in Down syndrome using mixed clinical and plasma biomarker measures with machine learning
Source: Alzheimers Dement. 2025 Jul 19;21(7):e70446. doi: 10.1002/alz.70446 (PMC12276070; doi:10.1002/alz.70446)
Supplement: Supplementary file 4 — Supporting Information [file ALZ-21-e70446-s004.docx]

**Supplementary Table 1.** Cognitive tests and outcome measures used in the event-based model.

| **Test** | **Description** | **Outcome measures used in this analysis** |
| --- | --- | --- |
| Cambridge Neuropsychological Test Automated Battery Paired Associate Learning (CANTAB PAL) task (36) | Participants observe and recall pattern locations to test visuospatial short-term memory. | First trial memory score 0-26, the number of times a subject chose the correct pattern locations on their first attempt, calculated across all assessed trials. |
| Cambridge Neuropsychological Test Automated Battery Intra/Extra Dimensional Set Shift (CANTAB IED) task (36) | Participants were required to learn rules about which was the ‘correct’ of two presented patterns on a computer screen, with a rule change after six consecutive correct trials. | Number of stages completed: 0–9. |
| Tower of London (37,38) | Participants move beads on a board to match presented configurations to test working memory and planning. | Total score: 0-10. |
| Developmental NEuroPSYchological Assessment-II - visuomotor precision (NEPSY-II) car and motorbike (39) | Participants are timed as they traced car, and motorbike tracks (divided into squares), with a time limit of 180s for each track. Errors are defined as squares where the line went outside the track, there was a broken line due to pen lift, or squares not completed in the time limit. | Error scores and track times were used to determine overall scores for the car and motorbike tracks combined: 0-52. |

**Note:** Higher scores indicate better performance in all tests.
